# Microfiber Emissions from Functionalized Textiles: Potential Threat for Human Health and Environmental Risks

**DOI:** 10.3390/toxics11050406

**Published:** 2023-04-24

**Authors:** Aravin Prince Periyasamy

**Affiliations:** Textile and Nonwoven Materials, VTT Technical Research Centre of Finland Ltd., P.O. Box 1000, FI-02044 Espoo, Finland; aravin.periyasamy@vtt.fi

**Keywords:** toxic chemicals, textile functionalization’s, microfibers, microplastics, sustainable productions, nanomaterials

## Abstract

The growing worldwide population is directly responsible for the increased production and consumption of textile products. One of the key reasons for the generation of microfibers is the use of textiles and garment materials, which is expected to increase. The textile industry is responsible for the invisible pollution that is created by textile microfibers, which have been detected in marine sediments and organisms. The present review paper demonstrates that the microfibers discharged from functionalized textiles exhibit non-biodegradable characteristics and that a considerable proportion of them possess toxic properties. This is primarily attributed to the impact of textiles’ material functionalization on their biodegradability. The potential for these microfibers, which are released from textiles that contain a variety of dyes, toxic chemicals, and nanomaterials, to pose a variety of health risks to both humans and other living organisms is discussed in this paper. In addition, this paper covers a wide variety of preventative and minimizing measures for reduction, which are discussed in terms of several phases ranging from sustainable production through the consumer, end of life, domestic washing, and wastewater treatment phases.

## 1. Introduction

The most common and resilient modification to our Earth’s surface area is the debris of fiber fragments [1]. Since plastic was first manufactured in mass quantities in the 1950s, the demand for artificial polymers and plastics has rapidly increased, and the global production of these materials in 2021 was 391 million metric tons (MMT) [2]. Furthermore, this number is anticipated to increase to 589 MMT by 2050 [3]. As a result of their enormous production and consumption rates, plastic particles including micro and macroplastics have begun to accumulate in Earth’s atmosphere, Mount Everest [4], on coastlines, on the most distant islands, and in the deep sea [5].

**Figure 1:**
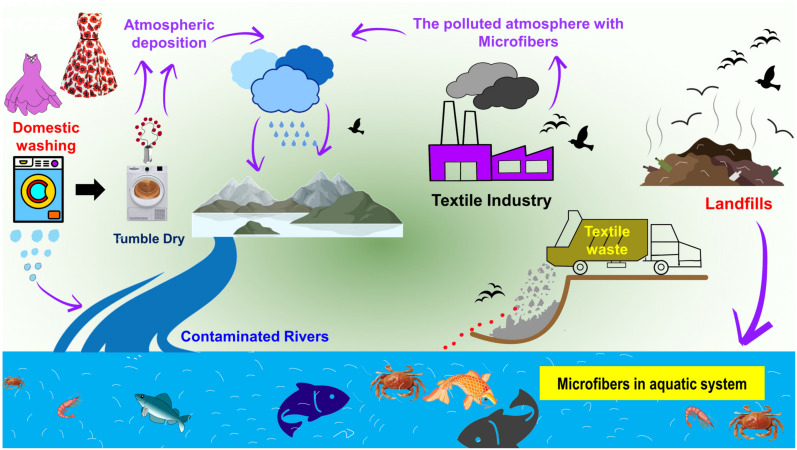
Main sources of microfibers in the aquatic environment from domestic washing, textile industries, and garment waste/landfills.

In the 1970s, microplastic pollution in the marine environment was discovered for the first time. Spherules, disks, and pellets were found floating on the surface of the Sargasso Sea [6], along the coasts of New England [7], in the surface waters of the Atlantic Ocean, and in the surface waters of the Pacific Ocean, respectively [8,9]. The phrase “microplastics” refers to fragments of plastic that are smaller than 5 mm in size. Microplastics are further divided into two categories, primary and secondary microplastics; this information has been clearly discussed in many recent articles [10,11,12,13,14,15,16]. Microplastics can stem from a range of resources consisting of synthetic textiles, tires, roadway markings, aquatic coverings, personal care/cosmetic products, and crafted plastic pellets, as well as from the gradual fragmentation of bigger plastics over time [17]; among these sources, the domestic washing of garments has the highest potential for the generation of microplastics [18,19,20,21,22,23,24] (Figure 1). In addition to petroleum-based plastic fibers, man-made cellulose fibers (e.g., viscose rayon) have also been detected in different environmental matrices of deep-sea sediment and macroinvertebrate fishes, thus increasing the interest of the scientific community in this kind of plastic pollution, which is usually underestimated [25,26,27]. Microfibers are similar to microplastics in terms of their size; they also measure less than 5 mm in diameter. However, their composition is not exclusively restricted to plastic. Fibers that originate from natural sources (e.g., cotton, wool, silk, and hemp) and plastic microfibers that originate from synthetic materials such as polyamide (PA), polyester, polypropylene, polyacrylonitrile (PAN), and polyethylene pose significant threats to the internal organs of the organisms that ingest them. Microfibers of natural origin typically exhibit biodegradability in the atmosphere. However, the functionalization of textiles can impede the biodegradation process, additionally, these materials are harmful to aquatic organisms.

Throughout its production process, the textile industry employs significant quantities of various chemicals [28]. Most of the polymeric additives that have been found in coastal environments are considered endocrine disruptors [29,30,31,32]. The manufacturing of fibers involves the addition of a large number of additives, the purpose of which is to enhance the fibers’ processability and functionality; a list of additives is provided in Table 1. For example, UV stabilizers in the form of benzotriazoles and benzothiazoles are frequently found in the effluent of municipal wastewater [33,34]. Titanium dioxide is widely used in synthetic fibers as a delustering agent to diminish the luster and transparency of yarns [35,36,37]. Diisobutyl phthalate is a plasticizer used in textile production that is reprotoxic, endocrine-disruptive, and toxic to aquatic life. To improve the flexibility and durability of textiles, phthalates are often added to polyvinylchloride (PVC)-based coatings [38,39]. To make flame-retardant textiles, hexafluorotitanate salts [40], TiO_2_ nanoparticles (NPs) [41,42] and brominated and phthalate compounds are commonly used in textile production, and these have shown reproductive and developmental toxicities [43,44]. Often, formaldehyde-based resins are added to improve the crease recovery properties of cellulose-based materials [45], and polyfluoroalkyl substances are added to improve the water repellency of textiles [46]. When it comes to natural fibers like cotton, it is possible to find hazardous pesticide residues that have been used during cultivation or applied for preservation purposes during storage [47]. Triclosan, which is extensively used in the garment industry as a fungicide, has been linked to endocrine disruption [48]. Furthermore, the process of coloring entails the utilization of dyes sourced from diverse chemical classes, predominantly comprising heavy metals that are commonly acknowledged as detrimental.

When textile microfibers are dumped into the ocean, they undergo photo- and biodegradation in addition to physical aging processes [49]. This ultimately results in the production of plastic debris at the micro- and nanoscale [50,51]. Polymer degradation is also regarded as a key source of dissolved organic carbon (DOC) release [52], as this DOC contains oligomers with varying degrees of oxidation [53] and various polymer additives (i.e., for man-made polymers) such as processing additives, enhancing additives, and functional additives [54].

**Figure 2:**
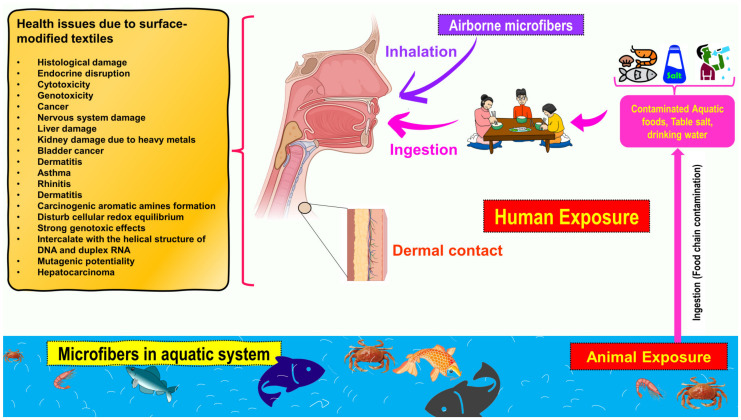
Schematic representation of exposure to microfibers through three routes: ingestion, inhalation, and dermal contact. Additionally presented are potential health risks of microfibers for human health via the food chain and dietary exposure.

Figure 2 illustrates how numerous routes of exposure might lead to microfibers accumulating in the human body. The textile industry is responsible for the invisible pollution that is created by textile microfibers, which have been detected in marine sediments and organisms [16]. This review covers some environmental routes (water, air, and soil) of microfiber contamination into the food web, describes their effects on human health, and presents new and relevant studies on their occurrence, fate, and behavior. This review paper reveals that the microfibers emitted from textiles are not biodegradable and that the functionalization of cellulose-based textile materials significantly influences their biodegradability. It is possible that these microfibers, which are produced from textiles that include a variety of dyes, hazardous compounds, and nanomaterials, could pose a variety of health threats to both human beings and other living organisms that are discussed in this work.

## 2. Textile Functionalization as the Source of Microfiber Toxicity

**Figure 3:**
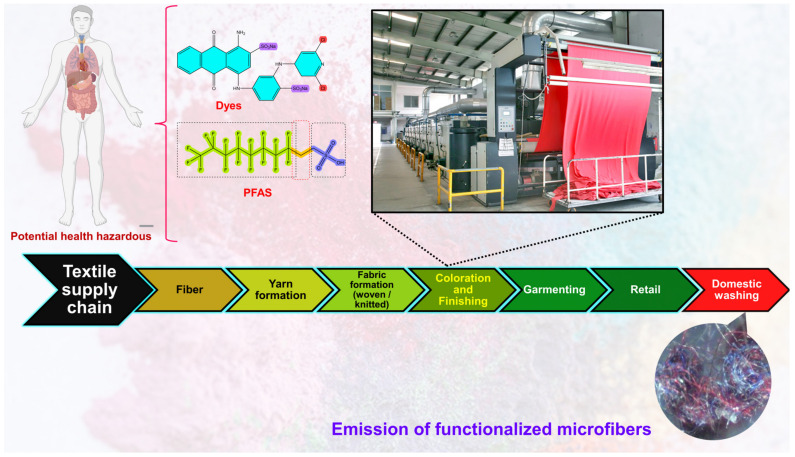
Textile production chain (i.e., linear chain).

**Figure 4:**
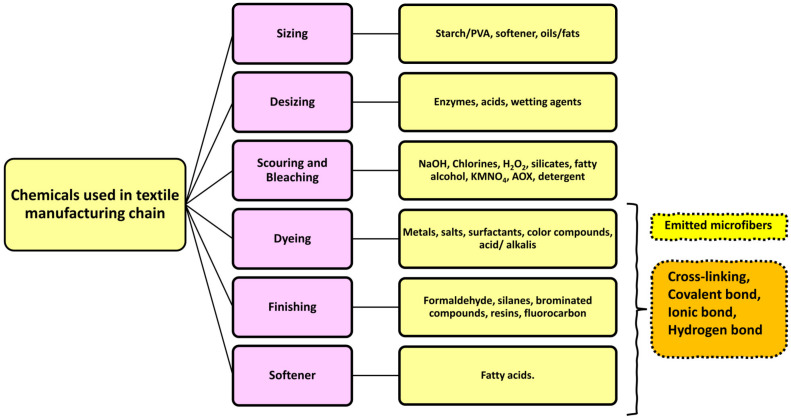
Various chemicals are used in the textile production chain.

The coloration and finishing process is one of the important and value-adding processes in the textile production chain (Figure 3). Typically, synthetic dyes and chemicals are functionalized with textile materials to improve some of their properties. [55]. Additionally, chrome or mordant dyes and metal complex dyes are utilized to achieve bright and dark colors. Most dyes contain heavy metals, including lead (Pb), arsenic (As), chromium (Cr), nickel (Ni), copper (Cu), cadmium (Cd), mercury (Hg), and zinc (Zn) (Table 2) [55,56,57]. Generally, heavy metals with a density of greater than 5 mg/cm^3^ are considered to have a high density. These heavy metals are non-biodegradable and difficult to clean up due to their complicated chemical makeup [58]. Consequently, the microfibers released from these textile materials contain heavy metals, which have carcinogenic, toxic, and nonbiodegradable effects that, in turn, cause enormous environmental problems [59,60,61]. Additionally, the metals are coated on the surface of the fibers to produce conductive textiles for electromagnetic shielding applications [62,63,64,65]. These heavy metals are notorious for their toxicity and negative effects on human health, as well as their impact on the environment. Additionally, the existence of organic additives, inorganic additives, and traces of monomers, metals, or other chemicals that can be discharged is a source of pollution that is more hazardous to human health than the released microfibers themselves [66,67]. The various chemicals used in the textile production chain are well described in Figure 4.

The purpose of the scouring step in the pre-treatment process is to make a textile material highly and uniformly absorbent, and it is carried out in alkaline conditions. Scouring removes practically all contaminants, except for natural pigments, that can be removed by either oxidizing or reducing conditions. However, the industry largely uses H_2_O_2_, in which atomic oxygen, superoxide anions, and hydroxyl ions perform the bleaching action in a process also known as the oxidation of natural color [68].

**Table 2:** Main use of heavy metals as additives in polymer products and their effects on human health.

Heavy Metals	Additives	Type of Polymers	Effects on Human Health	References
Antimony (Sb)	Flame retardants and biocides	Polyester cotton or polyester wool fabric	Metal-estrogenic effects and breast cancer	[69,70,71]
Aluminum (Al)	Stabilizers, inorganic pigments, and flame retardants	Polyester cotton or polyester wool fabric	Metal-estrogenic effects and breast cancer	[69,70,71,72]
Zinc (Zn)	Heat stabilizers, flame retardants, anti-slip agents, and inorganic pigments	Polyvinyl chloride (PVC), polyethylene (PE), and polypropylene (PP)	-	[71,72]
Bromine (Br)	Flame retardants	Polybutylene terephthalate (PBT), PE, polystyrene (PS), and PP	Apoptosis and genotoxicity	[71]
Arsenic (As)	Biocides	PVC, low-density polyethylene (LDPE), and polyesters	Congenital disabilities; lung, skin, liver, bladder, and kidney carcinogenic effects; gastrointestinal damage; and death	[71,72,73]
Lead (Pb)	Heat stabilizers, UV stabilizers, and inorganic pigments	PVC and all types of plastics in which red pigments are used	Anemia (less Hb), hypertension, miscarriages, disruption of nervous systems, brain damage, infertility, oxidative stress, and cell damage	[71,72,73,74,75]
Titanium (Ti)	UV stabilizers and inorganic pigments	PVC	Cytotoxicity on human epithelial lung and colon cells	[71,72,73,76,77]
Chrome (Cr)	Dyes for silk and metal complexes	PVC, PE, and PP	Allergic reactions to the body; nasal septum ulcer; severe cardiovascular, respiratory, hematological, gastrointestinal, renal, hepatic, and neurological effects; and possibly death.	[78]

### 2.1. Coloration

Dyes are a type of organic colorant that can be found in a variety of applications, ranging from food additives to the textile industry. In the industries that deal with textiles (paper, food, leather, paint, pharmaceuticals, and cosmetics), more than ten thousand distinct colors are employed. For example, direct dyes, reactive dyes, vat dyes, and cationic dyes are utilized for cellulose-based fibers, whilst acid and metal-complex dyes are utilized for wool and silk fibers [79,80].

In the last few decades, there have been a variety of environmental problems caused by the textile dyeing industry due to the emission of colored pollutants into the environment [81,82]. However, natural dyes have poor and low binding affinities; metals such as copper, hydrated potassium aluminum sulfate (alum), ferrous sulfate, tin, and chrome, are used as mordants to retain the natural dyes with the fibers. On the other hand, a growing interest in the use of natural dyes can be attributed to the recent rise in environmental consciousness, but some natural dyes require mordants due to their poor affinities. Commonly used mordants include ammonium alum (Al_2_(NH_4_)_2_(SO_4_)_4_·24H_2_O), soda alum (Al_2_Na_2_(SO_4_)_4_·24H_2_O), chrome alum (Cr_2_K_2_(SO_4_)·24H_2_O), ferric alum (Fe_2_(NH_4_)2(SO_4_)·24H_2_O), potassium dichromate, ferrous sulfate (FeSO_4_·7H_2_O), Cupric sulfate (CuSO_4_·5H_2_O), Stannous chloride (SnCl_2_), Stannic chloride (SnCl_4_·5H_2_O) [83]. Because of their low affinities, natural dyes are unsuitable for use on an industrial scale [84].

Based on their chemical composition, these dyes can be classified into azo, nitro, nitroso, indigo, phthalein, triphenylmethane, and anthraquinone groups. Azo dyes are the most common type of dye, accounting for 70% of all dyes used in the industry [85]; there are around 2000 varieties of azo dyes utilized in the industry, and over a million tons are produced annually [86]. Azo dyes degrade into aromatic compounds such as azoamine, which are toxic in nature.

#### 2.1.1. Vat Dyes

**Figure 5:**
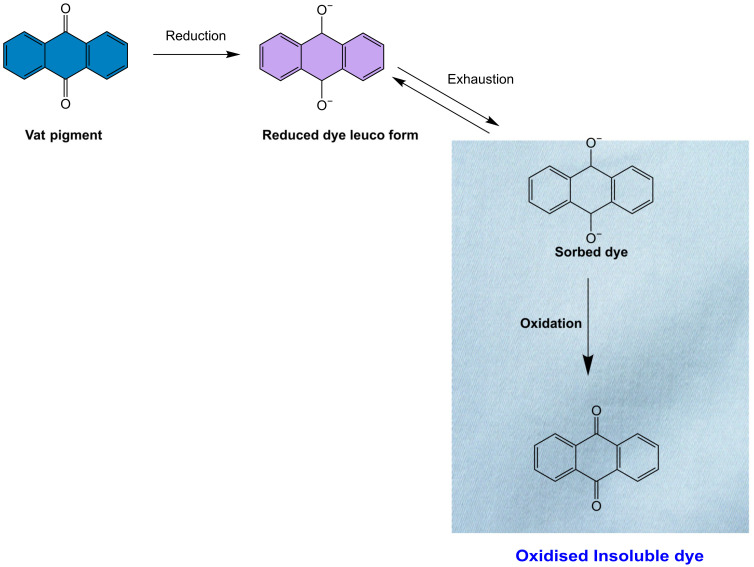
Mechanism of vat dye on the surface of cellulose.

Vat dye is particularly effective in coloring cellulose fibers, and it has excellent fastness against various agents such as detergent, bleach, and light due to its insoluble nature in water [61,87,88,89] (Figure 5). Indigo, a natural dye extracted from the Indigofera plant, is the best example of a vat dye [90,91].

#### 2.1.2. Sulphur Dyes

Sulphur dyes are extensively used to color cellulose-based fibers, and sulfur black is especially well-known [92]. Sulphur dyes containing functional groups such as hydroxy, nitro, and nitroso groups are produced using various aromatic compounds such as benzene and azobenzenes.

#### 2.1.3. Acidic Dyes

In an acidic atmosphere, acidic dyes are mostly utilized for nylon, wool, and silk (pH range of 3–7) [93]. Some acidic dyes include metal complexes that, if leached, pose health hazards [34].

#### 2.1.4. Disperse Dyes

Synthetic fibers often involve the use of disperse dyes because they target hydrophobic substances [94,95]. Since they are either insoluble or only partially soluble, most of these dyes are left in water baths, which results in a significant amount of wastewater being produced. Since these dyes do not contain ions, an aqueous dispersion may be made using them, and they are often applied to substrates such as polyester, nylon, acrylic fiber, and cellulose acetate [94,95,96].

#### 2.1.5. Reactive Dyes

**Figure 6:**
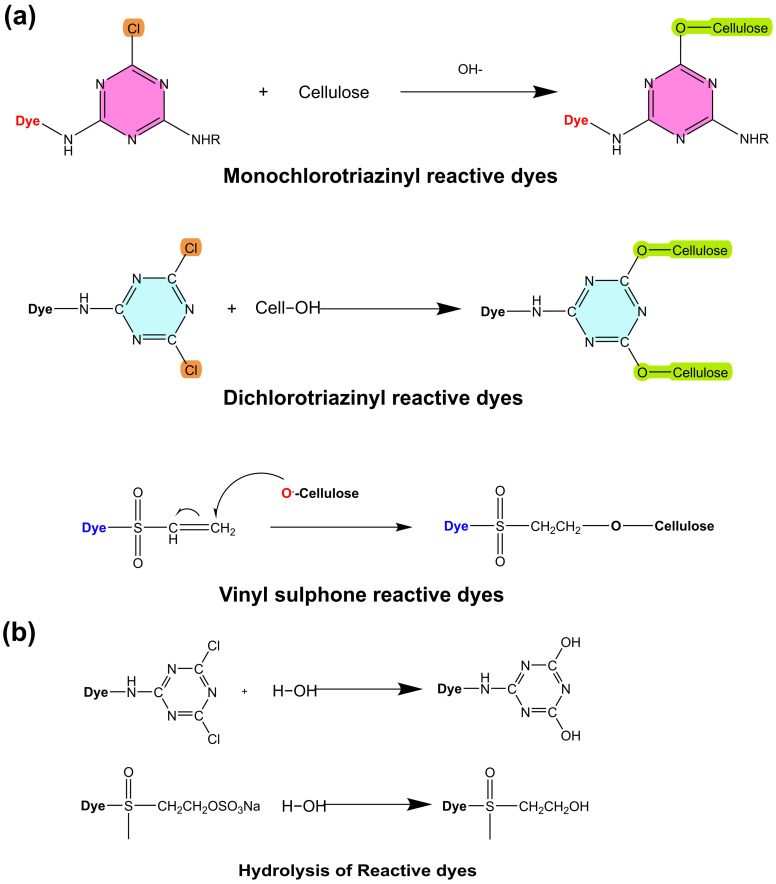
The chemical reaction of cellulose with different reactive dyes (**a**) the hydrolysis of reactive dyes during the dyeing process (**b**).

Reactive dyes are widely used for the coloration of textile substrates, especially cellulose. They mainly have two parts, a color group that is called a chromophore and a reactive functional group attached to the chromophore. These functional groups help dye molecules solubilize in water and form covalent bonds with cellulose during the dyeing process (Figure 6a) [97]. The dyeing process requires the use of an enormous quantity of salt, which raises the overall amount of dissolved salt in the effluent and, as a result, increases the amount of pollution in the water [98,99]. There have been many attempts to reduce the amount of salt and other chemicals during the dyeing process. Cationic reactive dyes have recently been developed and are promising candidates for the salt-free dyeing of cotton [100,101]. Cationic reactive dyes have a positive charge when dissolved in water due to attached functional groups [102]. Azo- and anthraquinone-based cationic dyes were synthesized, and the results showed that the exhaustion and fixation of cationic reactive dyes were better than those of normal reactive dyes [103]. The hydrolysis reaction of the reactive dye during the dyeing process is shown in Figure 6b.

### 2.2. Surface Modification and Finishing

In order to achieve desired effects, such as softness and aesthetics, and some functional properties such as flame retardancy, water repellence, and crease resistance, the finishing of textile goods involves the application of chemical and physical treatments. In general, the finishing process can be completed by applying the appropriate chemical mixture to the textiles using one of several processes (such as padding, spraying, coating, printing, or laminating) and then subjecting the textiles to the drying and curing stages of the process [38,104,105].

#### Grafting Functionalization (Chemical Treatment or Plasma Treatment)

**Figure 7:**
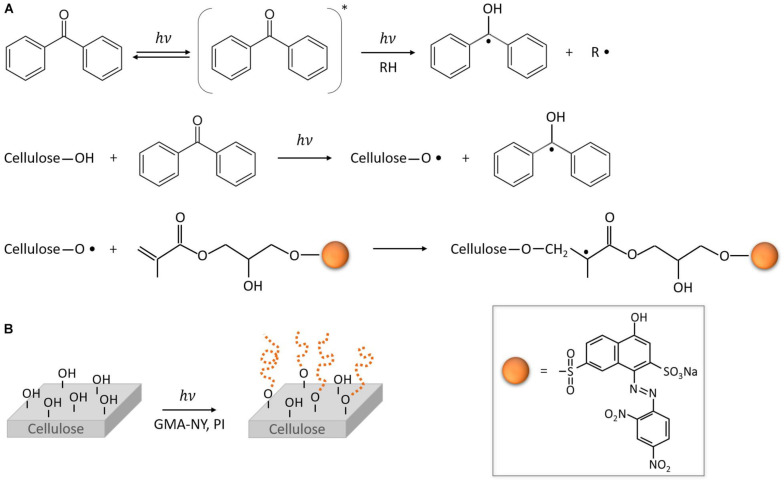
The mechanism of the photo-grafting reaction of GMA-NY onto a cellulose-based material (**A**); scheme of the photo-grafting process of a cotton substrate by light irradiation in the presence of a functionalized GMA-NY dye and a photoinitiator (**B**) (reprinted from [106] with Creative Commons Attribution License (CC BY)).

Grafting is one technique that can be used to increase the functionalization of cellulosic fibers. In cellulose, methacrylate moieties (GMA), such as poly(glycidyl methacrylate), are most commonly utilized [106,107,108]. For instance, a description of the photo-grafting mechanism of the modified halochromic GMA-NY dye onto a cellulosic material is shown in Figure 7 [106].

The ocular, digestive, respiratory, and cutaneous systems have all been shown to be sensitive to GMA’s harmful effects. Most human exposure takes place in the workplace, but it can also occur through the consumption of contaminated food. Even though there are no data available on the carcinogenicity of GMA, the presence of carcinogenic potential in the nasal cavity can be reasonably anticipated. Additional research is required to evaluate GMA exposure in human subjects [109,110].

### 2.3. Hydrophobization

**Figure 8:**
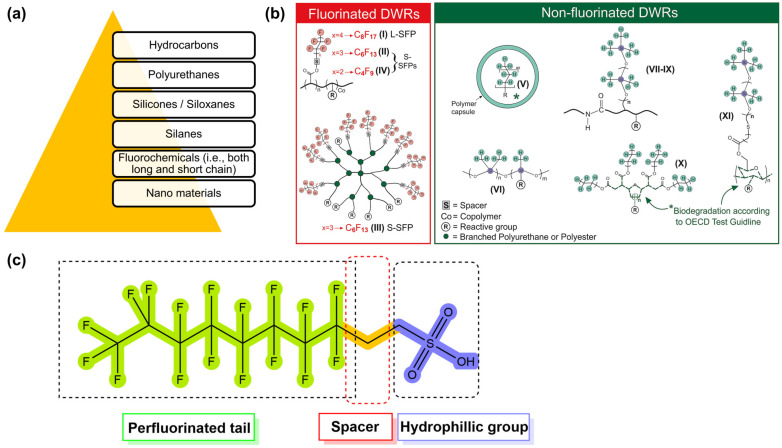
Different types of water repellents and waterproofing agents (**a**), simplified structures of fluorinated and non-fluorinated durable water repellents (DWRs) (**b**), and schematic PFAS molecule (**c**). Reprinted with permission [114]. Copyright 2021, Elsevier.

Fabrics can be treated to make them hydrophobic and repel water while still allowing air to pass through via a combination of chemical and/or physical techniques during the finishing process known as water-repellent finishing. Depending on how well something resists water and detergents, hydrophobicity can be either temporary or permanent [111,112]. Many different types of water repellents are available (Figure 8a), but the majority of the textile industry uses C8 fluorochemical compounds and silane-based chemicals [113].

**Figure 9:**
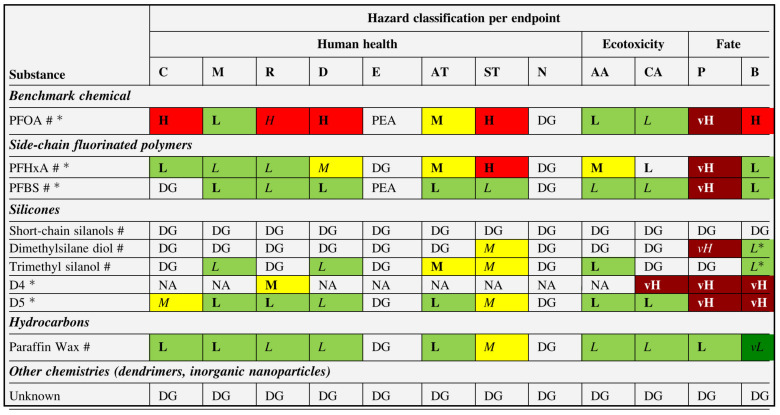
Hazard assessment results for selected water-repellent formulations. Note: Hazard classification abbreviations are: vL, very low; L, low; M, moderate; H, high; vH, very high; PEA, potentially endocrine-active; DG, data gap. Classifications in italics are of low confidence, and classifications in bold of high confidence. Classifications based on estimated data are marked with an asterisk (*). Reprinted with permission ([117]). Copyright 2021, Elsevier.

The toxicity of fluorochemicals is determined by their fluorocarbon chain length. Fluorochemicals are polymers with a perfluoroalkyl moiety (C_n_F_2n+1_) that are employed in water-repellent and waterproofing compositions [115,116]. They are commonly referred to as perfluoroalkyl and polyfluoroalkyl substances, or PFAS [117]. Almost half of all PFAS that are used are employed in the process of manufacturing water-repellent textiles on a global scale [46]. However, due to increased environmental consciousness in both the production and consumer phases, the usage of PFAS has decreased in recent years. In the production of water-repellent textile surfaces, novel, non-fluorinated DWR alternatives have recently been developed (Figure 8b). Figure 8c provides a condensed and simplified representation of a PFAS molecule. The hydrophobic nature of the fluorinated tail of a PFAS molecule is the distinctive quality that lends these compounds their extraordinary utility in the context of water-repellent applications. Long-chain PFAS remain in the environment, bioaccumulate in wildlife and humans, and have been demonstrated in laboratory animals to be reproductively, developmentally, and systemically hazardous [117]. Perfluorooctanoic acid (PFOA) is a chemical of concern that has been extensively studied, and alternatives were subjected to safety evaluations. Authors assigned data gaps for each hazard endpoint for dendrimers and inorganic nanoparticles because of insufficient formulation disclosure and/or hazard data, which are summarized in Figure 9; water repellents based on hydrocarbons were found to be the most eco-friendly, followed by those based on silicone and side-chain fluorinated polymers [117].

### 2.4. Crosslinking (Crease-Resistant)

**Figure 10:**
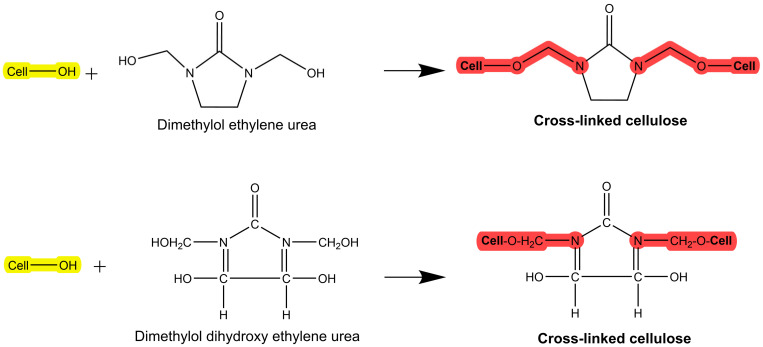
Crosslinking of formaldehyde-based resin on cellulose.

An easy-care or crease-resistant finishing can be applied to cellulose-based fabrics, providing resistance to crease formation and enhanced wet and dry wrinkle recovery [45]. The mechanism of such a finish is the inhibition of the easy movement of the cellulose chains by crosslinking with resins/polymers (Figure 10). Urea derivatives such as urea–formaldehyde and melamine–formaldehyde resins are commonly utilized in the industry [45]. Although the Government Accountability Office (GAO) has not discovered solid data on the severity of this problem, allergic contact dermatitis is the primary health risk associated with formaldehyde in textiles [118]. There are no definitive statistics on how many people may be sensitive to formaldehyde, although studies have revealed that some people develop allergic skin reactions to it, as reported by the GAO.

### 2.5. Fire-Retardant Finishing

Among the many different types of functional finishings that can be applied to textile substrates, flame-retardant finishing are essential because of their direct relationship to the risks to human health. Flame-retardant finishings of textiles can be classified as nondurable (i.e., borax and boric acid mixture, diammonium phosphate, and urea), semidurable (i.e., chlorine- and bromine-based halogen compounds), or durable (i.e., phosphorous- and halogen-based) depending on the effectiveness of the finishing in terms of performance following multiple washing cycles [38,119]. Among these chemicals, some of the brominated, chlorinated, and halogen-based flame retardants have been proven to be endocrine disruptors that can damage human health if ingested or inhaled [73]. The presence of organophosphate flame retardants, often known as organophosphorus flame retardants, is regularly found in both the environment and biota [120]. Tris-(2-chloroethyl) phosphate (TCEP), tris-(2-chloroisopropyl) phosphate (TCPP), tris-(1,3-dichloro-2-propyl) phosphate (TDCPP), tris-(2-butoxyethyl) phosphate (TBEP), triphenyl phosphate (TPP), and tricresyl phosphate (TCP) were examined in Danio rerio, and it was suggested that a wide variety of substances, including nuclear receptors, have the potential to disturb the endocrine system. To this day, very few reports have been compiled concerning the nuclear receptor activation of organophosphorus flame retardants [121].

### 2.6. Other Surface Modifications

#### 2.6.1. Cationization

One of the great triumphs of cationization is the elimination of salt from conventional reactive dyeing processes. The dyeing of cationic cotton with reactive dyes can reduce the quantity of salt in textile effluents by 70% [122,123]. Several authors have studied cotton cationization as an alternative for lower cost or more sustainable dyeing [99,123,124,125]. 3-Chloro-2-hydroxypropyl trimethylammonium chloride [122,123,126,127,128,129,130], poly diallyldimethylammonium chloride [131], 3-chloro-2-hydroxypropyl dimethyldodecylammonium chloride [132], and 3-chloro-2-hydroxypropyl dimethyloctadecylammonium chloride) [132] are the most studied cationized reagents. Of the various cationizing agents, 3-chloro-2-hydroxypropyl trimethylammonium chloride has been used at the industrial level [123]. 3-Chloro-2-hydroxypropyl trimethylammonium chloride (CHPTAC) has the following UN GHS classifications: H336, may cause drowsiness or dizziness; H351, suspected of causing cancer and has potential carcinogenicity; and H411 and 412, toxic to aquatic life with long-lasting effects [133].

#### 2.6.2. Nanomaterials

Nanotextile materials have recently demonstrated significant potential for use in a variety of advanced applications. During several process stages, such as fiber manufacturing, nanoparticles can be introduced to textiles by mixing in the polymer prior to fiber spinning, which improves the nanomaterials’ uniform distribution within the fiber volume. In most situations, nanoparticles are included during conventional fabric finishing processes such as dipping, padding, printing, and coating. Organic polymers are commonly employed to ensure nanoparticle adherence to textile surfaces [77,134]. Table 3 displays the various types of nanomaterials utilized within the textile industry.

Generally, nanoparticles intermingling in fiber molecular structures can be leached out when the fiber is subjected to the abrasion process. The leaching of nanomaterials is associated with high health risks and severe environmental threats. In case of conventional fabric finishing, the stability of nanomaterials on a fabric surface depends on the used organic polymer and its adherence properties. The stability of these nanomaterials depends on various marine conditions such as the temperature and salinity of water. Degradation results in a reduced molecular weight for the microfibers and possible cracks on the surface of fibrous materials. Because of the use of chemicals including nanoparticles and the textiles themselves, this situation raises further worries about the process’s potentially dangerous effects on both the environment and human health. Nanomaterials have the potential to interact with marine living species in one of three ways: (i) assimilation through contact with chemicals in the dissolved water phase, (ii) assimilation through the food chain, or (iii) release from microfiber debris that has been swallowed. However, the leaching of nanomaterials from textile materials depends on a number of factors, including the degree of binding, the type and shape of nanoparticles, the type of fabric, and the aging of the functionalized fabric. Nanoparticles discharged from textiles into the air, water, and landfill must also be considered because of their potential to either directly or indirectly damage human health [77,135].

**Table 3:** Different nanomaterials used in the textile industry.

Nanomaterial	Properties
Silver (Ag)	Antibacterial (odor) and electrical conductivity
Titanium Dioxide (TiO_2_)	UV-protective, self-cleaning, water-repellent, and soil-repellent
Zinc Oxide (ZnO)	UV-protective, antibacterial, self-cleaning, abrasion-resistant, and stiffness
Silicon Dioxide (SiO_2_)	Water-repellent, dirt-repellent, and abrasion-resistant
Aluminum Oxide (Al_2_O_3_)	Abrasion-resistant and flame-retardant
Nanoclays (e.g., montmorillonite)	Abrasion-resistant, flame-retardant, and active ingredient support

## 3. Implication of Microfiber Contamination on Human Health

There are multiple ways in which microfibers can enter the body of a human being, including ingestion [14,136], inhalation [137], and skin contact [138], as is well-described in Figure 2. Each of these ways of being exposed is in some way connected to a certain environment and the chemical–physical features of that environment. One of the most significant sources of airborne microfibers is the textile production chain, which includes spinning, weaving, processing, landfilling, waste incineration, and the drying of clothing after washing [18,139] (Figure 2).

Since inhalation is one of the primary methods through which humans are exposed to microfibers, the released microfibers that contain many additives, such as nanomaterials and other chemicals such as perfluoroalkyl derivatives and formaldehyde, pose significant risks to human health. Although no research has been published on this topic, some studies have investigated the effects of polystyrene nanoparticles (PS-NPs) on human lung epithelial [140,141,142,143,144]. PS-NPs may directly interfere with membrane transporter activity in A549 cells, affecting xenobiotic and endogenous substrate disposition.

The microfibers emitted from functionalized textiles that have toxic properties might adversely affect fish and other aquatic life [145], which has the potential to create serious problems [146,147]. These microfibers have raised concerns since they have the potential to impact animal populations, which are essential for maintaining ecosystems [148,149], and the animal populations that provide vital ecosystem services might be harmed by reactions with these microfibers [150]. It is recognized that foodstuffs are the main sources of microfibers for humans (Figure 2) since most food, such as table salt [151,152], drinking water [153,154,155,156], beer [157,158], fruits/vegetables [159,160], and canned fish [161], is contaminated with microfibers. A recent study [162] demonstrated the presence of micro and nanoplastics in a variety of foods, including apples and carrots, which were shown to have the highest levels of contamination. Additionally, the authors observed microfibers in carrots (1.51 µm) and lettuce (2.52 µm), which had the largest amount [162]. Overall, 52,600–307,750 of microfibers were discovered in vegetable samples, whereas 72,175–130,500 were discovered in fruit samples [162]. In addition, Cauwenberghe and Janssen [136] found that the average consumer of shellfish in Europe consumes 11,000 microplastics per year. According to research [14], the average American diet and lifestyle result in the consumption of microfibers that is estimated to range between 39,000 and 52,000 particles per person. However, different age groups, genders, geographical conditions, and individual dietary habits and lifestyles all affect the amount of consumption. It is challenging to assess the actual threat that microfibers pose to human health based on the data that are currently available regarding the presence of microfibers in a wide variety of food sources and the corresponding findings of toxicity tests. More work needs to be performed to develop an analytical method that is both standardized and operational for identifying and quantifying microfibers, and more research needs to be conducted to investigate the potential effects that microfibers and associated chemical contaminants could have on human health [14,163].

In most cases, dermal contact with microfibers is related to exposure to monomers and additives, which are on a long list of endocrine disruptors. However, this route of exposure is regarded to be less significant [138,164,165]. For instance, research was conducted on the dermal uptake of substances in rainbow trout, showing evidence for the uptake of 1 μm latex spheres from the water in the surrounding environment, with particles localizing and remaining in the surface and sub-surface epidermal cells of the skin, as well as in phagocytes underlying the surface of the gills [164].

In addition, oxidative stress can be caused in human epithelial cells when they are exposed to microplastics and nanoplastics [166]. The term “persorption” refers to the mechanical kneading of solid particles (with a diameter of up to 130 μm) in the gastrointestinal tract, where they pass through gaps in the single-layer epithelium at the villus tips and into the circulatory system. This process is thought to be a possible route of uptake in the digestive tract [167,168].

Recent research conducted by Nur et al. [169] resulted in the development of a probabilistic lifetime exposure model for the purpose of determining the amount of microplastics consumed by children and adults. This model considers microplastics consumed through inhalation, eight different food types, intestinal absorption, biliary excretion, and plastic-associated chemical exposure through a physiological-based pharmacokinetic submodel. Based on biphasic, reversible, and size-specific sorption kinetics, the chemical absorption results of the food and ingested microplastic of all nine intake media revealed that the contribution of microplastics to overall chemical intake was negligible. Considering the need for future research, discussions regarding the currently unknown contributions of different types of foods should be held. We will likely trust the results of the aforementioned studies for the time being, as it may take some time for microplastics to reach humans due to the complexity of the food system, but the probability of ingestion is growing as microplastic production and consumption continue to rise.

**Figure 11:**
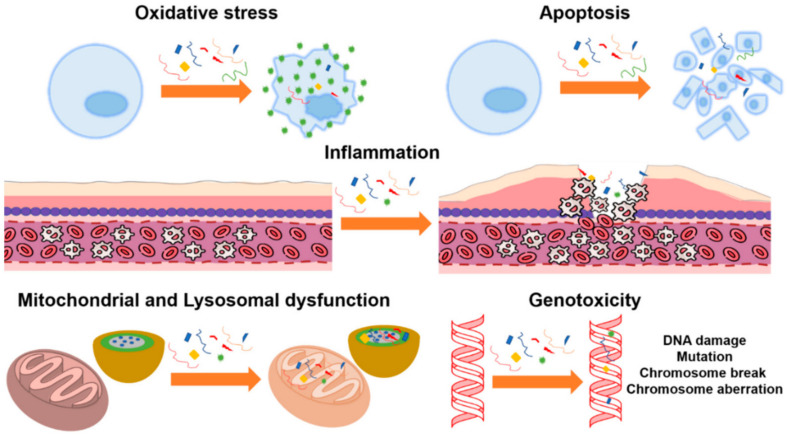
Toxicological effects of polystyrene microparticles on cell cultures: oxidative stress, apoptosis, inflammation, mitochondrial and lysosomal dysfunction, and genotoxicity, (Reprinted from [174] with Creative Commons Attribution License (CC BY)).

Potentially harmful consequences of microplastics on human health [137,139], including inflammation and subsequent genotoxicity [170], have been identified. Similar to other non-biological micro- and nanoparticles, inhaled microplastics can translocate into the pulmonary epithelium through diffusion, direct cellular penetration, or active cellular absorption [171]. Interstitial fibrosis and granulomatous lesions were observed in the lungs of employees who work in the plastic industry, and these issues were attributed to acrylic, polyester, and nylon dust [137,139,170]. Microfiber absorption via inhalation has been compared to that via ingestion (via the food web) in the published literature [172]. Human microfiber ingestion is rather low compared with exposure levels, with studies finding that microfibers are breathed in between 3 and 15 times more than they are ingested [173,174]. Numerous toxicological investigations of ingested microfibers have been published in the scientific literature. Most of this research employed polystyrene particles as a benchmark material for more sophisticated microfibers, and only a few studies concerned polyethylene [174,175,176,177,178,179]. In addition, the dose, dose rate, and period of exposure employed in the trials all had significant impacts on the harmful consequences. Most studies showed toxicological effects on parameters such as oxidative stress [180,181,182,183,184,185], inflammation [185,186,187,188,189,190,191], mitochondrial dysfunction [192,193,194,195], lysosomal dysfunction [196,197], and genotoxicity [198,199,200]. Figure 11 illustrates the main toxicological effects found in cell cultures.

Arif et al. [201] collected the stools of human fishermen living in the coastal area of Surabaya, Indonesia. They found that more than 50% of the studied samples included a microfiber concentration ranging from 3.33 to 13.99 µg per gram of stool, and most of them were high-density polyethylene (HDPE). Additionally, Philipp et al. [144] studied microfibers in human stools, and they observed PP and PET with a size range of 50–500 μm and a concentration of 2 particles per gram of stool. Additionally, a median of 20 microfibers with size range from 50 to 500 μm per 10 g of human stool was observed by Yan et al. [190].

**Figure 12:**
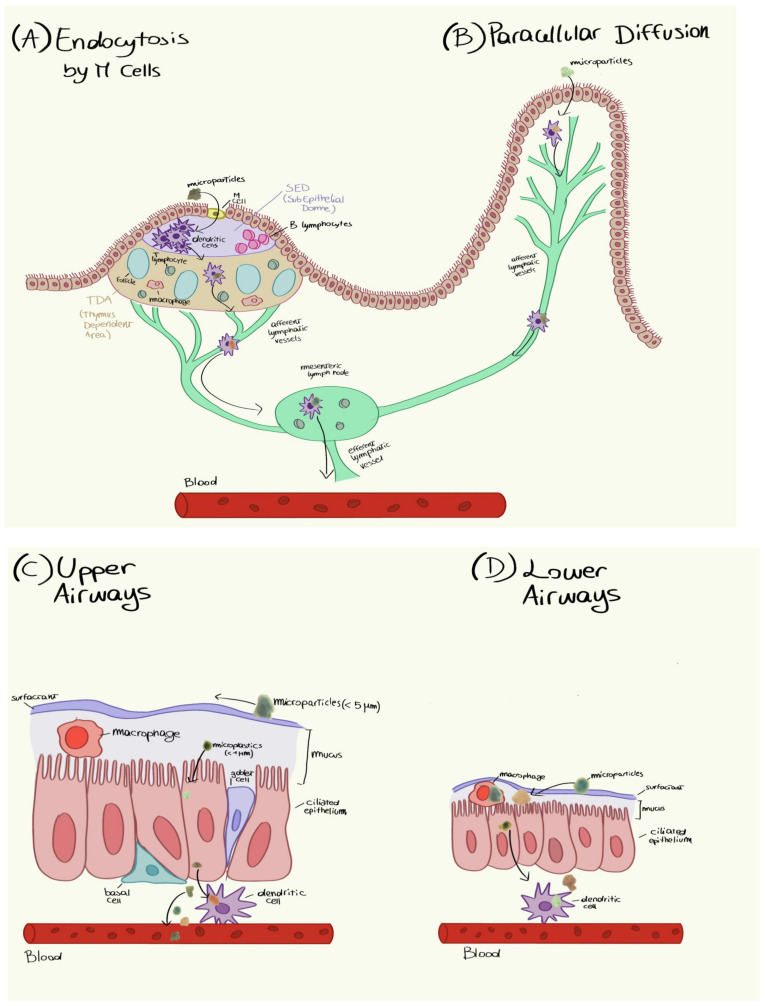
Hypothetical mechanisms by which microfibers penetrate human tissues. Reprinted with permission ([202]). Copyright 2021, Elsevier.

Ragusa et al. [202] found microfibers in human placentas. The particles were found in the placentas of four healthy women who had normal pregnancies and births. Microfibers were detected on both the fetal and maternal sides of the placenta and in the membrane within which the fetus developed. Unfortunately, we do not know how microfibers reach the bloodstream or if they come from the respiratory or the gastrointestinal systems. Figure 12 shows the possible entry and transport mechanisms of microfibers from the respiratory and gastric organs to the placenta. Under microspectroscopy, 12 microfiber fragments were isolated in four human placentas. In particular, five microfibers were found on the fetal side, four were found on the maternal side, and three were found in the chorioamnionitis membranes, indicating that once inside the human body, these microfibers can reach placenta tissues at all levels. It is noteworthy that small portions of placentas (~23 g with respect to a total weight of ~600 g) were analyzed, letting us hypothesize that the number of microfibers within an entire placenta is much higher.

As these findings show, it is important to continue looking for microfibers in human fluids, and additional research should be conducted to determine how microfibers interact with and make their way into the human body. The contamination of samples by airborne microfibers is an important aspect of the microfiber detection process that needs to be considered. Therefore, a significant amount of focus needs to be paid to the treatment of samples to prevent the incorrect identification of microfibers in human samples, particularly for particles of less than 10 mm in size, and to increase the total number of samples that are gathered. In addition, there is an immediate need for more research that is conducted on a transnational and interdisciplinary scale and focuses on the toxicology of these particles to fully understand the effects that these particles have in the long term on humans and to assist health organizations in developing prevention guidelines.

## 4. Positive Actions toward Reductions in Microfibers

**Figure 13:**
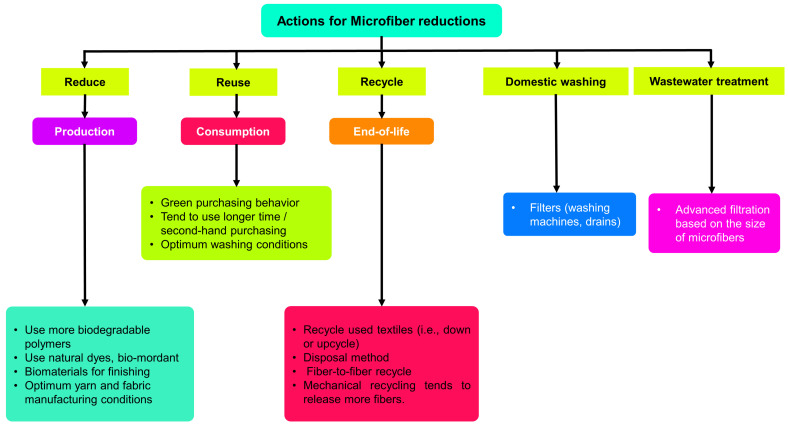
Positive actions toward avoidance of and reductions in microfibers.

The most important thing to do to reduce microfiber pollution is to adopt preventative steps to avoid and limit the creation of microfibers from the very earliest stages of textile manufacturing and usage. Figure 13 depicts positive activities that can be undertaken to avoid and minimize the development of microfibers.

### 4.1. Sustainable Production

In recognition of the significant quantity of textiles produced annually, it is imperative to guarantee the proper management of microfibers extracted from textile garments throughout the entire manufacturing process spanning from fiber to garment [203]. The production of fibers and fabrics that are more durable, enabling longer durations of use and reuse, and that reduce microfiber emissions in day-to-day activities such as wearing and washing is one potential approach to addressing the problem [55,204]. Creating a sustainable and circular bio-economy may require using natural cellulosic materials and producing biobased and biodegradable textile materials. Currently, the following fibers are the most discussed alternatives to existing polymers: PLA [205], polyhydroxyalkanoate (PHA) [206], polycaprolactone (PCL) [207], polybutylene succinate (PBS) [208], polyhydroxybutyrate (PHB) [209], Ioncell [210], BioCelsol [211], Infinna [212], and Renewcell [213]. Ioncell, BioCelsol, Infinna, and Renewcell are regenerated cellulosic fibers, while PLA, PHA, and PHB are renewable and biobased thermoplastics. However, it appears that not all solutions presented here are suitable for use in textiles or resolving the issue of microfiber emissions. This is a result of the fact that biodegradable plastics do not decay in the same manner under all environmental conditions, which is a problem that will call for greater research in the not-too-distant future. Many biodegradable polymers are created in a lab, but few are commercially viable due to cost, processing, and mechanical properties.

PLA has various limitations, particularly a poor heat resistance, when utilized in textile applications; however, research is underway to develop improvements. The manufacturing capacity of bioplastics has increased, and it is anticipated to grow by 7.6 million metric tons (MMT) by 2026 [214]. However, only 10% of these polymers are used in textile applications; the rest of them are used in packaging and other applications [215]. One of the most essential factors to consider when evaluating biopolymers is the production process’s environmental impact. Life cycle assessments (LCAs) of biopolymers have revealed their advantages over petroleum-based polymers in terms of climate change and energy use [216,217]. Despite the benefits described above, bioplastics cannot replace all synthetic and existing textile fibers.

Other processes, such as dyeing and finishing, also need to be modified, and the use of more natural-based materials, such as natural dyes and biobased materials, could help reduce the toxicity of the microfibers that are being emitted. Additionally, it is also recommended that pre-treatment techniques such as prewashing decrease the amount of fibers released during each washing cycle and that adequate filtering systems are available at locations where products are manufactured.

Since ancient times, natural dyes have been used to color food, leather, wood, and natural fibers including wool, silk, cotton, and flax. Natural dyes are extracted from roots, bark, leaves, flowers, and fruits (Table 4). Due to environmental consciousness, textiles using non-toxic and eco-friendly natural dyes are important. Most natural dyes require a mordant to fix onto cellulose or other textile fibers. In earlier times, metal-based mordants were used to color textiles with natural dyes, but they caused environmental [218,219,220] and health [219] problems due to their high contents of metal ions [221], which leach during domestic washing. As a result, the utilization of bio-mordants may constitute an optimal alternative to the utilization of mordants based on metals [222,223]. Overall, natural dyes are renewable, biodegradable, and skin-friendly, and they may offer health advantages [84,223,224,225,226].

The creation of bioactive textiles heavily relies on biopolymers, which are polymers obtained from biological sources. Polysaccharides, chitosan, and sericin proteins can replace chemical-based textile finishing agents. Biopolymers have many benefits, including low costs, biodegradability, and biocompatibility. Due to their useful features, such as antibacterial, UV-protective, fragrance finishing, insect-repellent, and flame-retardant properties, polysaccharides have attracted a lot of attention for usage in textiles [227,228]. Antimicrobial properties have been added to cotton, polyester, and wool textiles via chitosan finishing [229,230]. The ability of cyclodextrins (CDs) to create inclusion complexes with other compounds via host–guest interactions has shown promising results in textile finishing. CDs are porous, so they can hold many different types of molecules, and they can be chemically crosslinked to form chains that behave like polymers [231]. Biocompatibility, biodegradability, resistance to ultraviolet light, antibacterial activity, and the ability to absorb moisture are only few of the intrinsic features of sericin, a natural protein. The performance of synthetic fibers was enhanced using sericin [232]. Recent research has revealed that sericin can be used to add antibacterial properties to the finishes of natural fibers such as cotton and wool [232,233]. Regarding biocompatibility, nontoxicity, and bioactivity, alginate is another sustainable biopolymer, and it is commonly used as a wound treatment and in the finishing of textiles to impart antibacterial characteristics [234,235,236,237]. Herbal extracts provide aromatic, antibacterial, skin-nourishing, and mothproofing properties. Aloe vera, neem, grape, mulberry fruit, banana stem, citrus oil, sandalwood oil, jasmine, and lavender are considered sustainable finishing agents for textile finishing. Microencapsulated aroma therapy finishes on textiles were found to reduce chemo and radiotherapy side effects for cancer patients [238]. Apart from their aroma, these extracts activate the immune, nervous, psychosocial, cell regeneration, antibacterial, and anti-depressive systems.

**Figure 14:**
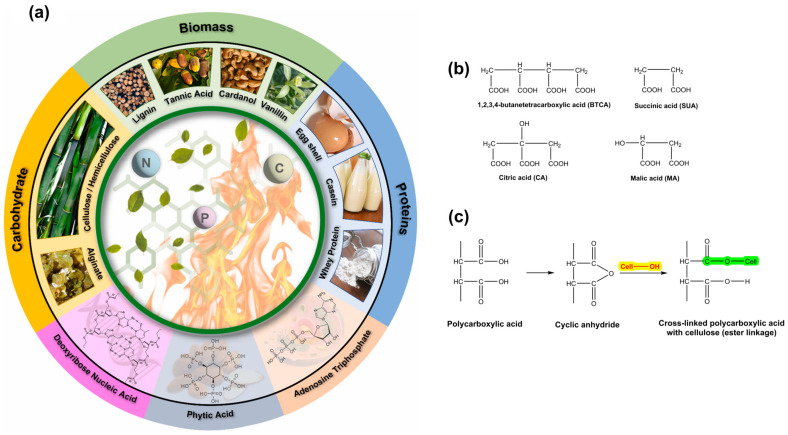
Bio-derived flame retardants (**a**), various polycarboxylic acids used for the crease resistance finishing of cotton (**b**), and crosslinked celluloses with polycarboxylic acid (**c**). Reprinted with permission ([239]). Copyright 2022, Elsevier.

Numerous biobased additives have been utilized in the production of textiles to impart flame retardancy. These include hemicellulose, alginate, lignin, tannic acid, cardanol, vanillin, casein, whey protein, eggshell, deoxyribose nucleic acid, phytic acid, and adenosine triphosphate [239]. The utilization of different biobased additives for imparting flame retardancy to textiles is illustrated in Figure 14a. Melamine–formaldehyde resins are widely utilized as crosslinking agents in anti-wrinkle finishing processes due to their exceptional efficacy and economic advantages. Nevertheless, this type of N-methylol compound has the potential to emit carcinogenic and toxic formaldehyde. Growing environmental apprehension has necessitated the development of crosslinking agents that are more environmentally sustainable. Polycarboxylic acids, including 1,2,3,4-butanetetracarboxylic acid (BTCA), malic acid (MA), and citric acid (CA), have been the subject of extensive research for several decades due to their potential as formaldehyde-free compounds (Figure 14b) [45,240,241,242]. The hydroxyl groups of the cellulosic chains in cotton are esterified by polycarboxylic acids via the creation of cyclic anhydride intermediates with five or six members (Figure 14c).

### 4.2. Consumption Phase

Overall, Europe will generate over 15 kg of textile waste in 2022, and 85% of textile waste comes from household garment waste and is not properly reused [243]. Educational campaigns are urging customers to use clothes longer to reduce fast fashion. Textile manufacturing initiatives should support these approaches to improve apparel quality and durability. Another step is streamlining pre-owned clothing delivery and sales. Since microfibers shed less with each wear and wash cycle, selling and using pre-owned products may reduce their release, export rates, and littering. Optimizing synthetic textile washing to prevent abrasion can also reduce shedding. Optimization can reduce detergent use, water use, and washing speed.

### 4.3. End of Life, Recycling and Disposal Phase

Increasing textile waste collection and reuse can overall reduce the amount of landfills and possibly reduce microfiber emissions. Used textiles can be reused into industrial rags, furniture decorations, purses, backpacks, advertising textiles, and more to extend their lives. Thus, a process for disposing and processing discarded textiles must be devised and publicized [244]. The chemical recycling of polymers (i.e., both cellulose and thermoplastic polymers) needs development to increase recovery and conversion efficiency, as well as to reduce material characteristics and quality [245,246,247,248]; however, energy and pollution issues must be addressed. Some European countries, especially Finland and Sweden, have developed several regenerated cellulose fibers for upscaling. Converting the paper and pulp business to regenerated cellulose fiber is easy. The market for regenerated cellulose fibers is expected to rise by 10.5% a year by 2026, reaching 7.1 MMT by 2021 [203,211,249].

### 4.4. Domestic Washing and Wastewater Treatment Phase

Recent research has found that 87% of microfibers in washing effluents may be retained by an integrated stainless steel filter with a 150–200 µm mesh [19]. Better filters in washing machines can also help reduce fiber pollution, so most washing machine companies provide a separate filter that can reduce microfiber emissions [250,251]. The use of laundry balls designed to catch fibers during washing has also been reported to reduce fiber counts in washing effluent by 26%. It has also been suggested that microfiber emissions could be reduced by using washing bags to enclose synthetic textiles during laundering and to trap fibers from garments’ hems. When comparing the overall efficiency of different approaches used to decrease fiber emissions, it is crucial to consider the mesh or pore size, filtering capacity, and capture capacity of filters. Sand filtration, membrane bioreactor (MBR) treatment, or pile fabric filtration can reduce microfiber emissions from wastewater treatment plants. Stockholm’s wastewater treatment plants are implementing the world’s largest MBR to renovate a piece of their sludge system. Using granular activated carbon and other technologies to remove micropollutants from water can reduce microfiber emissions (particularly 50 μm) by up to 61% [252,253]. However, this type of technical progress may require more electricity, chemicals, and other resources, as well as a large initial investment and continuing maintenance costs. Currently, many countries lack the infrastructure needed to improve wastewater treatment plants. The pre-treatment of sludge can be effectively used to minimize microfiber contents. In recent years, plastic collectors have been employed to collect floating plastic garbage and prevent it from being moved downstream.

## 5. Conclusions

The textile industry is responsible for the undetectable pollution caused by microfibers that are emitted from textile garments, which have been found in the sediments of marine environments and in marine creatures. The functionalization of textile materials requires various toxic additives including dyes, chemicals, and nanomaterials. As a result, the functionalization of textiles and the microfibers emitted in those textiles, which can be dangerous to human health and lead to a host of environmental problems, was the primary emphasis of this review. This paper reviewed the various functionalization processes and different toxic chemicals, dyes, and nanomaterials involved in the textile production chain, and their influence on microfiber emissions were discussed. For instance, the effects of the different heavy metals present in dyes (such as lead (Pb), arsenic (As), chromium (Cr), nickel (Ni), copper (Cu), cadmium (Cd), mercury (Hg), and zinc (Zn)), various toxic chemicals used in the functionalization of textiles (such as polyfluoroalkyl substances used to provide water-repellent properties, chlorine, bromine, phosphorous, and halogen-based chemicals used to provide flame-retardant properties), and various formaldehydes on crease-resistance properties were discussed. In addition to various nanomaterials such as Ag, TiO_2_, ZnO, SiO_2_, and Al_2_O_3_, nanoclays are used to functionalize textile materials. Therefore, the potential for these microfibers, which are released from textiles that contain a variety of dyes, toxic chemicals, and nanomaterials, to pose a variety of health risks to both humans and other organisms was discussed in this paper. Concerning the utilization of a variety of chemicals, numerous expected issues regarding these emitted functionalized textile microfibers were discussed, and the health issues associated with them were investigated. In addition, this paper covered a wide variety of preventative and minimizing measures for reduction in terms of several phases ranging from sustainable production through the consumer, end of life, domestic washing, and wastewater treatment phases.

## Figures and Tables

**Table 1:** Typical additives are used in man-made fiber production.

Type	Function	Examples
Processing Aids	Antioxidant	Hindered phenols, hindered amines, and phosphites
	Hydrolysis Stabilizer	Carbodiimide
	Nucleating Agent	Talcum powder, boron nitride, and organic phosphate salts
	Lubricant	Stearates and low-molecular-weight wax
	Polymer Processing Aid	Fluoropolymers
	Surfactant	Stearates and polyethylene glycols (PEGs)
Enhancing Additives	Plasticizer	Tributyl citrate and acetyl tributyl citrate
	Chain Extender	Difunctional acid derivatives, anhydrides, and epoxides
	UV Stabilizer	Hindered amine light stabilizers (HALS), titanium dioxide (TiO_2_), zinc oxide (ZnO), and carbon black
	Flame Retardant	Phosphorous derivatives, halogen derivatives, and HALS
	Thermal Protection	Zirconia
Functional Additives	Colorant	Pigments, dyes, and carbon black
	Delustering	TiO_2_, ZnO, mica, and optical brightening agents
	Antistatic	Carbon black, carbon nanotubes, graphene, and ZnO
	Antimicrobial	TiO_2_, ZnO, nano-sized metal particles (Ag^+^, Cu^2+^, Zn^2+^), plant extracts, and phenol
	Water/Oil Repellent	Silicone and fluorine compounds

**Table 4:** Common natural dyestuffs obtained from different vegetable regions.

Part of the Plants	Dyestuff
Root	Turmeric, madder, onions, and beetroot
Bark/Branches	Purple bark, sappan wood, shillicorai, khair, red, and sandalwood
Leaf	Indigo, henna, eucalyptus, tea, cardamon, coral jasmine, and lemon grass
Flowers	Marigold and kusum
Fruits/Seeds	Pomegranate rind, beetle nut, myrobolan, and latkan

## Data Availability

Not applicable.
